# Workplace Adaptive Technologies for Working Adults With Cognitive and Physical Disabilities

**DOI:** 10.1093/geroni/igaa057.3176

**Published:** 2020-12-16

**Authors:** Patricia Heyn

**Affiliations:** University of Colorado Anschutz Medical Campus, Aurora, Colorado, United States

## Abstract

Individuals with disabilities usually have difficulty in finding and maintaining employment prospects and thus, they are extremely underrepresented in the workforce. These challenges are even greater when the person has both cognitive and physical disabilities. While there is evidence supporting the benefits of employing individuals with disabilities in the workforce, employers are usually unprepared to hire individuals with disabilities. They are also concerned that the work productivity may be impacted by the employee with a disability. Thus, technology can play an important role in helping a person with cognitive and /or physical impairment work on tasks that require memorization and assembly performance. We will present a mobile technology system that was planned and piloted with working adults with physical and cognitive impairments. Founded on our pilot study, mobile technologies hold the potential to help people with disabilities to perform jobs that require memorization as well as systematic assembly tasks.

